# The Impact of Phenotype of Inflammatory Bowel Diseases, Inflammation Activity and Therapy on Mucosal Mature Cd83^+^ Dendritic Cell

**DOI:** 10.3390/jcm13072070

**Published:** 2024-04-03

**Authors:** Bruna Rošić Despalatović, Marija Babić, Andre Bratanić, Ante Tonkić, Žarko Ardalić, Katarina Vilović

**Affiliations:** 1 “J&J MEDICI” Polyclinic for Internal Medicine, 21000 Split, Croatia; bruna.rosicdespalatovic@gmail.com; 2 Medical School, University of Split, 21000 Split, Croatia; marija-babic92@hotmail.com; 3 Department of Gastroenterology and Hepatology, University Hospital Split, 21000 Split, Croatia; ante.tonkic@mefst.hr (A.T.); gastro_zavod@kbsplit.hr (Ž.A.); 4 Department of Pathology, University Hospital Split, 21000 Split, Croatia; patologija_zavod@kbsplit.hr

**Keywords:** CD83^+^ dendritic cell, ulcerative colitis, Crohn’s disease

## Abstract

**Background:** Crohn’s disease (CD) and ulcerative colitis (UC) are well-defined phenotypes of chronic inflammatory bowel diseases (IBDs). A mechanism of inflammation in these diseases is partially controlled by the intestinal dendritic cell (DC). In this study, we observed a mature CD83^+^ DC in colonic bioptic samples, and its correlation with disease phenotype and activity. **Methods:** The study included 219 subjects: 100 with UC, 44 with CD and 75 healthy subjects. Colonic biopsy specimens were incubated with the primary antibody Anti-CD83. Intraepithelial CD83^+^ DCs were counted per 100 enterocytes. The presence of CD83^+^ DC was analysed according to the type of IBD, histopathologic inflammation activity and treatment outcome. **Results:** The presence of mature CD83^+^ DCs (0, ≥1) differed according to disease types of IBD (*p* = 0.001), histologic inflammation activity (*p* = 0.049) and applied therapy (*p* = 0.001). The odds for CD83^+^ DC presence were 5.2 times higher in the CD group than in the control/UC group. The odds for CD83^+^ DC presence were 2.6 times higher in subjects without inflammation or chronic inflammation than with acute inflammation. They were also 3.7 times higher in subjects without therapy. The cut-off value 0.5 CD83^+^ DC (Rock analysis area = 0.699; SE 0.046; *p* < 0.001; 95% CI: 0.609–0.788) had been assessed as a differentiation marker between UC and CD. **Conclusion:** Presence of CD83^+^ DC could be used as a possible parameter in distinction between UC and CD, as well as a predictor of inflammation activity and treatment outcome.

## 1. Introduction

Crohn’s disease (CD) and ulcerative colitis (UC) represent the two main phenotypes of inflammatory bowel diseases (IBDs), which are characterized by chronic inflammation of the entire or of specific parts of the gastrointestinal system. Both phenotypes share similar pathophysiologic mechanisms and clinical presentation. The real etiopathogenesis is still incompletely understood [1]. It is known that genetic predisposition, along with microbiologic intraluminal factors and environmental factors, determines the onset as well as the course of the disease [2,3]. This fine mechanism of regulation of chronic inflammation is, at least in part, controlled by the intestinal dendritic cell (DC) [4]. This cell controls, “feels” and catches an intraluminal antigen and transports it to the lymphatic tissue [4]. Therefore, its role is balancing the response to the luminal antigen [4]. Disturbing this fine balance between the tolerability and the active immunologic response is the crucial step in IBD pathogenesis [5,6]. DC is the main population of antigen-presenting cells in lamina propria [7,8,9]. Besides lamina propria, these cells are present in lymphoid aggregates of the small intestine [10]. In specimens of colonic and rectal biopsies, an immature HLA-DR^+^lin^−^ DC of CD11c^+^ subpopulation has been identified, which, through maturation, obtains the phenotype of CD83^+^ mature DC [11,12].

At present, the widely accepted pathophysiologic model of the role of DC proposes that immature DC continuously enters the mucosal lamina propria, Peyer’s patches and lymphoid follicles of the colon. It becomes more mature and positions itself in different parts of the colon, depending on the expression of specific cytokines [13,14]. After transporting the antigen through epithelial cells, or uptaking apoptotic particles of the epithelial cells, it travels to the mesenteric lymph nodes or intrafollicular regions in the form of an activated, mature cell. This migration is associated with cytokine receptors for the T zone of the lymph node, such as CCR7. This ‘ready’ but still inert DC can stimulate the differentiation of a T cell into a regulatory T cell, which, in turn, mediates as a tolerant observer after the encounter with the antigen. Intestinal stromal cells can also create a suppressive environment which stimulates DC to steer a differentiation of T lymphocytes into regulatory T lymphocytes. Contrary to this response to “innocent” antigens, mucosal pathogens initiate active local and systemic immunologic responses. The initial contact of a pathogen with epithelial cells, its cell components, adherent DC or macrophages include recognizing microbial pathogen-associated molecular patterns (PAMPs) or pattern recognition receptors (PPRs), such as TLR receptors [15,16,17,18]. TLR signalling from epithelial cells results in production of inflammatory cytokines and chemokines such as IL-1, IL-8, IL-6, TNFα, CCL5 and CCL20, which attract and activate neutrophils, macrophages and DC [19]. Beside this intermediate pathway, DC can be activated directly by pathogens through TLR and other surface receptors. This causes a complete cell activation with a high level of expression of MHC, costimulatory and adhesive molecules and cytokines. A phenotype of the consequent T lymphocyte response is either directly or indirectly determined by the type of host–pathogen contact. Signals which DC receives directly from pathogens and tissue signals on the site of contact in lymph nodes during the first contact of “priming” T lymphocytes will activate specific DC subpopulations (characterized by the expression of specific PPR), which then determine the type of response to the pathogen: either toleration or active defence response [20,21,22,23]. It is evident that the function of mucosal DC and its subpopulations is regulated by the local microenvironment which includes immunologic cells as well as luminal bacteria [24]. All these factors take part in maintaining intestinal homeostasis. However, certain functions of DC and its subpopulations still remain unknown, and further studies are warranted to obtain more information regarding factors which regulate intestinal inflammation.

The aim of this study is to assess the impact of applied therapy, level of inflammation in biopsy specimens and phenotype of inflammatory bowel diseases on the presence of mucosal mature CD83^+^ dendritic cell in colonic biopsy samples.

## 2. Methods

### 2.1. Subjects

In this study, there were 219 subjects included, all older than 18. They underwent colonoscopy during a two-year period, from 2015 to 2017, at Gastroenterology clinic of Polyclinic for internal medicine, gynaecology and psychiatry “J&J MEDICI”. The subjects were divided into three groups: (1) subjects with IBD diagnosis, currently without therapy; (2) subjects with IBD who have taken azathioprine or anti-TNF therapy for at least 6 months but have not taken corticosteroids for at least 6 weeks; (3) healthy subjects in the control group. The diagnosis of UC was based on ECCO guidelines from 2008, 2012 and 2018 [25,26,27]. The CD diagnosis was based on ECCO guidelines from 2010, 2016 and 2018 [27,28,29].

There were 100 subjects with UC. Among them, 68 were without therapy, 15 on azathioprine and 17 on anti-TNF. There were 44 subjects with CD. Among them, 19 were without therapy, 13 on azathioprine and 12 on anti-TNF. In the control group, there were 75 healthy subjects.

### 2.2. Endoscopy

Colonoscopy was performed with the VP 3500HD endoscopic video processor with an XL 4450 light source and EC530 and EC600 endoscopes (Fuji, Tokio, Japan). In UC subjects, Mayo endoscopic subscore (MES) was used as an index of endoscopic assessment of disease activity. This score evaluated for the macroscopically most severely inflamed part of the colon, scoring erythema, vascular pattern, friability and erosions. [30,31] In CD subjects, the simple endoscopic score for Crohn’s disease (SES-CD) was used as an index of endoscopic assessment of disease activity. This score calculates ulcer size, proportion of the surface area that is ulcerated, proportion of the surface area affected and stenosis [31,32,33].

### 2.3. Faecal Calprotectin

The faecal calprotectin level was measured in stool samples once in each subject during the ten-day period prior to his/her colonoscopy. Calprotectin concentration (µg/g) was assessed by the immunoturbidimetric method (Buhlmann laboratories AG, Schonenbuch, Switzerland) on the device Beckman-Coulter AU 168 (Beckman Coulter Inc., Brea, CA, USA).

### 2.4. Colonic Biopsy Samples Analysis

Colonic biopsy samples of subjects with UC and CD were taken from sites of, endoscopically, the most active disease.

(a) Histologic analysis

Bioptic samples of colonic mucosa were fixated in 4% buffered formaldehyde for 24 h, then washed in 0.1 M phosphate buffer and, after dehydrating, embedded in paraffin at 56 °C. They were cut in 4 μm wide slices and attached to positively charged slides (Superftost plus, Thermo Scientific, Waltham, MA, USA). For staining, standard hemalaun-eosinophilic (HE) was used in the automatic staining device HE 600 (Ventana, Tucson, AZ, USA). Stained samples were analysed using a light microscope Olympus BX41 (Olimpus, Tokio, Japan).

Histopathologic results were divided in three groups: acute inflammation, chronic inflammation and no inflammation.

(b) Immunohistology analysis

**Figure 1:**
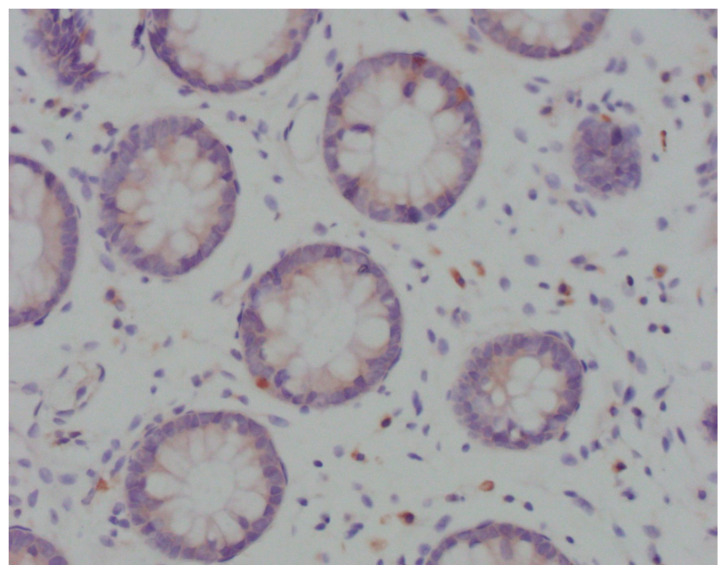
Light microscopy of CD83^+^ DCs (Olympus BX41; magnification ×40). Cytoplasmically brown-stained, irregularly shaped, CD83^+^ cells in close contact with the crypt epithelium.

Immunohistochemical analysis was performed in the device Bench Mark ULTRA IHC/ISH Staining Module (Ventana, Tucson, AZ, USA) with positive control. After deparaffinization in xylol and rehydration through alcohol descending concentrations, the slices were cooked in EDTA pH 8.2 buffer for 10–30 min. Endogenous peroxidase was inactivated by incubation in H_2_O_2_. After that, the slices were washed out in phosphate buffer solution (PBS) and incubated in the primary antibody Anti-CD83 ab205343 (Abcam, Cambridge, UK) in a moist atmosphere for 32 min. After washing out in PBS, the slices were incubated in a system for secondary detection Ultraview Universal DAB DEtection Kit (Ventana, Tucson, AZ, USA). Stained samples were analysed using the light microscope Olympus BX41. In areas with the most intensive staining, intraepithelial mature CD83^+^ DCs were counted per 100 enterocytes (Figure 1).

### 2.5. Statistic Analysis

All data analysis was performed with SPSS 20. As the Shapiro–Wilk test indicated statistically significant deviation from the normal distribution of all numeric variables, the median and interquartile ranges were used. Statistical significance of the differences in categorical demographic and clinical characteristics was calculated by the chi-square (χ^2^) test and Fisher’s exact test. Analysis of the statistical significance of differences in CD83^+^ DC number among the three study groups was performed with the Kruskal–Wallis’s test. Post hoc analysis was performed with the Mann–Whitney test. In our analysis, we also used binary logistic regression.

Statistical significance was set to *p* < 0.05, and all confidence intervals were given at 95%.

## 3. Results

### 3.1. All Subjects

There were 219 subjects included in this study, all older than 18. Among them, 100 (46%) had UC, 44 (20%) had CD and 75 (34%) were in the control group. There were 113 male (51%) and 106 (49%) female subjects. The median age was 40 years (Q1–Q3: 31–55 y; min–max: 15–80 y) (43.7 ± 15.6 y).

Groups with a different presence CD83^+^ DC (0, ≥1) were adjusted according to age (*p* = 0.889) and sex (*p* = 0.419) (Table 1).

There was a significantly different presence of mature CD83^+^ DCs (0, ≥1) according disease types (*p* = 0.001). In the CD83^+^ DC ≥ 1 group, the number of UC subjects was 1.6 times lower, and the number of CD subjects was 4.7 times higher than in the CD83^+^ DC = 0 group. The odds for CD83^+^ DC presence were 4 times higher in the CD group than in the control group (OR: 4; 95% CI: 1.1–14.6; *p* = 0.035). Analysing the number of subjects with UC and CD (without controls) in groups with different CD83^+^ DC presence (0, ≥1), we found a significant difference (0, ≥1) (*p* < 0.001). There were only 8% CD subjects without CD83^+^ DC. The odds for CD83^+^ DC presence were 7.8 times higher in the CD group than in the UC group (OR: 7.8; 95% CI: 2.2–27; *p* < 0.001) (Table 1).

The presence of mature CD83^+^ DCs (0, ≥1) significantly differed among subjects with a specific histologic inflammation type (*p* = 0.049). There were 1.7 times fewer subjects with acute inflammation in the CD83^+^ DC ≥ 1 group and 1.7 times more subjects with acute inflammation in the CD83^+^ DC = 0 group. The odds for CD83^+^ DC presence (CD83^+^ DC ≥ 1) in subjects without inflammation were 2.1 times higher than in those with acute inflammation (OR = 5.9; 95% CI: 1.1–4.3; *p* = 0.030). There was no significant difference between the number of subjects with acute and chronic inflammation (*p* = 0.086). But the odds for CD83^+^ DC presence were 2.2 times higher in subjects without or with chronic inflammation than in those with acute inflammation (OR = 2.2; 95% CI: 1.2–4.1; *p* = 0.015) (Table 1).

The presence of mature CD83^+^ DCs (0, ≥1) significantly differed among subjects according to applied therapy (*p* = 0.001). There were 3,6 times more subjects with CD83^+^ DS ≥ 1 than those with CD83^+^ DS = 0 in the group with therapy. The odds for CD83^+^ DC presence were 4.8 times higher in subjects who had been on therapy than in those who had not (OR = 4.8; 95% CI: 1.8–12.7; *p* = 0.002) (Table 1).

A multiple logistic regression was then performed, with CD83^+^ DC (0, ≥1) as the dependent variable and subjects’ groups (combined controls and UC; CD), histopathology (combined chronic and no inflammation; acute inflammation) and therapy (no; yes) as independent variables. All three variables showed statistically significant correlation with CD83^+^ DC (0, ≥1) in multivariate logistic regression. The odds for CD83^+^ DC presence were 5.2 times higher in the CD group than in the combined control/UC group. The odds for CD83^+^ DC presence were 2.6 times higher in subjects without inflammation or with chronic inflammation than in those with acute inflammation. They were also 3.7 times higher in subjects without therapy than in those on therapy (Table 2).

**Figure 2:**
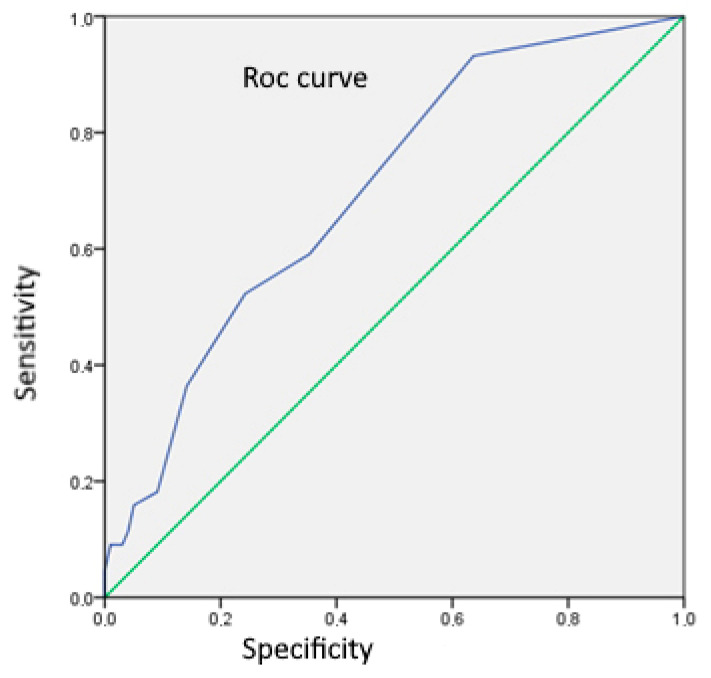
ROC analysis curve for assessment of number of CD83^+^ DC as a differentiation marker between UC and CD (area = 0.699; SE 0.046; *p* < 0.001; 95% CI: 0.609–0.788).

Using ROC analysis for the assessment of CD83^+^ DC number as a differentiation marker between UC and CD, we obtained a cut-off value of 0.5, with sensitivity and specificity of 93.2% and 36.4%, respectively (Figure 2).

Analysing the number of CD83^+^ DC according to the type of disease shows that only 7% subjects with CD had n CD83^+^ DS/100 e ≤ 0.5 (Table 3).

### 3.2. Ulcerative Colitis Subjects

The presence of mature CD83^+^ DCs (0, ≥1) was also analysed separately in the UC group (Table 4).

The presence of mature CD83^+^ DCs (0, ≥1) differed among subjects with different endoscopy scores (MES) (*p* = 0.002). The odds for DC presence (CD83^+^ DC ≥ 1) were 4.7 times higher in subjects with MES 0 or MES 1 than in subjects with MES 2 or MES 3 (OR: 4.7; 95% CI = 1.8–12.2; *p* = 0.002). The presence of mature CD83^+^ DCs (0, ≥1) also significantly differed among those with acute inflammation and those with chronic or no inflammation (*p* = 0.046). The odds for CD83^+^ DC presence were 2.6 higher in the chronic or no inflammation group than in the acute inflammation group (OR:2.6; 95% CI = 1.1–6; *p* = 0.013). There was no significant correlation between the presence of CD83^+^ DC (0, ≥1) and calprotectin level (*p* = 0.555). The presence of mature CD83^+^ DCs (0, ≥1) significantly differed according to whether therapy is applied or not (*p* = 0.007). We found 3 times more subjects with CD83^+^ DC ≥ 1 who had been on therapy (azathioprine, anti-TNF) than those who had not. The odds for DC presence were 4.5 times higher in subjects on therapy in comparison to those without therapy (OR: 4.5; 95% CI = 1.6–13; *p* = 0.006). Subjects’ groups with different CD83^+^ DC presence did not differ according to gender (*p* = 0.532) or age (*p* = 0.530) (Table 4).

A multivariate logistic regression confirmed the combined effect of MES and histopathologic results on CD83^+^ DC presence. The odds for CD83^+^ DC presence were 5 times higher in group with MES 0,1 and with no/chronic inflammation than in group with MES 2.3 and with acute inflammation (OR: 5; 95% CI 1.4–17.5; *p* = 0.012), adjusted for therapy (Table 5).

The presence of mature CD83^+^ DCs (0, ≥1) significantly differed among subjects’ groups on different therapy (*p* < 0.004). There was a statistically equal number of subjects without (46%) and with (54%) CD83^+^ DC among UC subjects who were not on therapy. The number of subjects without CD83^+^ DC in the group with no therapy was 2 times higher than in the control group, 3.5 times higher than in the group on azathioprine, and 2.6 times higher than in the group on anti-TNF (Table 6).

### 3.3. Crohn’s Disease Subjects

The Crohn’s disease group had only 3 subjects without CD83^+^ DS, 15 subjects with one CD83^+^ DS and 26 subjects with CD83^+^ DS = 2–10. Therefore, we divided them in two groups: CD83^+^ DS ≤ 1 (n = 18) and CD83^+^ DS > 1 (n = 26), and analysed them according to different features (Table 7).

The presence of mature CD83^+^ DCs (≤1, >1) significantly differed between different endoscopy scores SES-CD (*p* = 0.006). The odds for CD83^+^ DC > 1 were 7.9 times higher in the SES-CD ≤ 5.9 group than in the SES > 5.9 group (OR = 7.9; *p* = 0.006). The presence of mature CD83^+^ DCs (≤1, >1) also significantly differed regarding calprotectin level. There were 3 times more subjects with CD83^+^ DC > 1 than CD83^+^ DC < 1 in the group with calprotectin value ≤ 449 µg/g (*p* = 0.006). The odds for CD83^+^ DC > 1 were 7.9 times higher in subjects with a calprotectin level ≤449 µg/g than in those with calprotectin level > 449 µg/g (OR = 7.9; *p* = 0.006).

In addition, the presence of mature CD83^+^ DCs (≤1, >1) significantly differed comparing the group with acute inflammation and the groups with no inflammation and chronic inflammation (*p* = 0.004). The odds for CD83^+^ DC > 1 were 8.4 times higher in the subjects’ group without or with chronic inflammation than in the group with acute inflammation (OR = 8.4; *p* = 0.003). Most of the subjects with CD83^+^ DC > 1 were on therapy. Subjects’ groups with different CD83^+^ DC presence did not differ according to gender (*p* = 0.176) or age (*p* = 0.879) (Table 7).

There was a significant difference in CD83^+^ DC presence (≤1; >1) between healthy subjects and CD subjects on any type of therapy (χ^2^ = 22.4; *p* < 0.001; Cramer’s V = 0.434). All the subjects on anti-TNF had CD83^+^ DC > 1. The number of subjects with CD83^+^ DC > 1 was 3.7 times higher in those on azathioprine than in those without therapy, and it was 1.6 times higher than in healthy subjects (Table 8).

## 4. Discussion

In this study, we have analysed the presence of CD83^+^ DC in colonic biopsy specimens of CD and UC patients, and we found that the presence was higher in CD than in UC subjects. There were few previous studies which analysed mature DC presence in different IBD types. Middel and Baumgart have proved an increased number of CD83^+^ DC in the tissue of patients with CD and UC. Their study showed the increased number of cells which expressed costimulatory molecules such as CD40, CD80, CD83 and CD86 in the mucous tissue of CD, but also in those of UC patients [34,35,36]. Radwan et al. had primarily compared both IBD types with the control group of healthy subjects, assessing the number of mucosal mature DC grown in cell culture; a significantly higher presence of mature DC was observed in both IBD groups compared with that of controls [37]. On the other hand, Radwan–Kwiatek’s studies of DC in blood showed a significantly decreased number of immature cells, which was explained as the result of cell migration into the inflamed intestinal tissue. This decrease in cell presence correlates well with the severity and extent of the inflammation process [38]. Baumgart et al. reported that in IBD patients, a low expression of costimulating molecule CD86 in DC in peripheral blood was found, and CD83 expression was absent [36]. Velde et al. reported similar results, as well as Middel et al., while Bell et al. in their study did not prove a statistically significant difference in CD83^+^ DC, regardless of the existing difference in the DC number median [11,12,34].

When we analysed subjects according to the presence of CD83^+^ DC in a specific histologic inflammation type, we found that this presence was higher in subjects with chronic inflammation or no inflammation than in those with acute inflammation. Additionally, there was significantly lower presence of CD83^+^ DC in the subjects’ group with histopathological signs of acute inflammation than in healthy controls. Until now, only Bates et al. reported results from a mice colitis model study, in which the role of CD83^+^ DC was studied at different levels of inflammation. Their results showed that loss of CD83 in DC would lead to the worsening and acutisation of the inflammation level in colitis model [39]. Middel et al., comparing the CD83^+^ DC number in areas of active vs. non-active inflammation of the same patient, found a higher number of CD83^+^ DC in samples with active inflammation [34]. In the study conducted by Bell et al., a difference in DC number median was found between different histopathologic levels of inflammation; however, it was not statistically significant [11].

Assessing the number of CD83^+^ DCs as a differentiation marker between UC and CD in our study, we obtained a cut-off value of 0.5 CD83^+^ DCs, with a sensitivity and specificity of 93.2% and 36.4%, respectively. We showed that only 7% of subjects with CD had n CD83^+^ DS ≤ 0.5. There are no earlier studies which assess DC number as a differentiation marker between UC and CD.

We also analysed a relationship between CD83^+^ DC presence and applied therapy in study subjects and found a significantly higher presence in those who were on therapy. In the study of Silva et al., the influence of different therapies on CD83^+^ DC number in patients with CD was analysed; the DC number was decreased significantly only in patients treated with systemic corticosteroids [40].

### 4.1. Ulcerative Colitis Subjects

According to our results, subjects with acute inflammation had significantly lower presence of CD83^+^ DC compared to those with chronic or no inflammations. In the previously mentioned study of Bates et al., a role of the CD83 molecule in the regulation of immunologic homeostasis was proposed: a loss of CD83 in DC had worsened the inflammation in the colitis model [39]. Their results were based on the mice colitis model [39]. It was also proved that DC isolated from lamina propria in mice significantly decreased the expression of the maturation marker CD83 [39]. This mice colitis model could give us an explanation of how DC, in immunologic reservoirs such as intestinal lamina propria, prevents excessive inflammation. An overexpressed CD83 on the mucosal surface “protects” from colitis, while loss of expression of CD83 in DC worsens the colitis. This was the first time that the role of CD83^+^ DC in immunologic homeostasis was proved [39]. In study of Kawashima et al. on surgical specimens from UC patients, an increased CD83^+^ DC number was isolated by the immunofluorescent method from cell culture, primarily from lymphoid aggregates in specimens with histopathologically active disease [41]. An increased number of CD83^+^ DC was found also by Baumgart et al., this time by isolation from blood by the immunocytochemical method [42].

In our study, most subjects in the group with an endoscopic index of disease activity MES 2 and 3 had histologically acute inflammation, accompanied with higher faecal calprotectin levels. Similar results were reported in the study of Roseth et al., who analysed histological inflammation activity in tissue specimens of UC patients, with the purpose of defining the calprotectin cut-off level in mucosal healing [43]. Results from the studies of Viera et al., D’Inca et al. and Kaiser et al. were also similar to our results; the highest level of faecal calprotectin was in subjects with acute inflammation [44,45,46]. Bodelier et al. reported similar results regarding MES grade and faecal calprotectin levels, as well as D’ Haens et al. [47,48]. A recent meta-analysis published by Moslia et al. showed similar results, with the overall sensitivity and specificity of faecal calprotectin level in predicting endoscopically active disease at 88 and 79%, respectively [49].

According to our results, the CD83^+^ DC presence was significantly higher in subjects’ groups with MES 0 and 1 than in MES 2 and 3. Duchmann et al. concluded, by indirectly assessing the infiltration of lamina propria with T lymphocytes in endoscopically actively inflamed parts of intestinal mucosa, that present mature DC were activators of T lymphocytes [50].

A combined influence of MES endoscopic index and histopathologic results on CD83^+^ DC presence was shown in our results. CD83^+^ DC presence was the highest in group MES 0.1/no inflammation/chronic inflammation. There are no earlier studies which compare both endoscopic and histopathologic UC activity with the presence of mature CD83^+^ DC.

Analysing the presence of CD83^+^ DC in UC patients’ group with different therapy, we found significantly lower presence of CD83^+^ DC in patients without therapy than in those on specific therapy (azathioprine or anti-TNF). When we additionally compared each of these UC patients’ groups (with specific therapy or without therapy) with the control group, we found the lowest CD83^+^ DC presence again in the UC patient group without therapy. Bhandaru et al. in their study proved in vitro the influence of azathioprine on migration of DC and releasing of TNF-L [51]. On the other hand, Hart et al., who were also assessing the effect of azathioprine on DC through TLR expression, did not find significant correlation between the number of DC and azathioprine therapy [52].

### 4.2. Crohn’s Disease Subjects

While conducting the study, we observed that the presence of CD83^+^ DC was significantly lower in the group with acute inflammation compared to those with chronic or no inflammations. Similar results were reported by Silva et al., after they had compared colonic tissue specimens of CD patients with inflammation with those without inflammation [53]. In their study on cell culture, Bell et al. observed that immediately after bioptic sampling, there was no significant difference in CD83^+^ DC number among groups with different histologic inflammation activity, although there was a difference in the median values of CD83^+^ DC in these groups. Middel et al. in their study on CD patients found an increased CD83^+^ DC number in specimens with histologically active inflammation, compared with those without inflammation [34].

Assessing the endoscopic index SES-CD, we found a significantly higher median of SES-CD in patients with histologically acute inflammation than in those with other inflammation levels. This was accompanied by higher levels of faecal calprotectin levels in these patients. Similar results were reported by Bodelier et al., as well as by D’ Haens et al. [47,48]. An overall meta-analysis of Mosli et al., showed similar results, with overall sensitivity and specificity of faecal calprotectin levels for predicting endoscopically active disease at 87 and 67%, respectively [49]. According to our results, the CD83^+^ DC presence was significantly higher in subjects’ groups with higher SES-CD calprotectin level. There are no earlier studies which compare endoscopic SES-CD score and calprotectin with the presence of mature CD83^+^ DC.

When we compared the CD83^+^ DC presence of subjects on therapy (azathioprine or anti-TNF) with that of the control group, we found that it was significantly higher in subjects on therapy. The median of CD83^+^ DC number was 3 times higher in the azathioprine group or anti-TNF group than in the control group. In the group of CD patients who were on anti-TNF, all subjects had a number of CD83^+^ DC > 1. These results indicate that there is a difference in the presence of CD83^+^ cells in the tissue samples, but they do not answer whether the presence of this cell has an impact on the response to therapy or whether it has predictive value for the course of the disease. Hart et al. also assessed the effect of anti-TNF drugs on DC through expression of CD40, and they found significant decrease in CD40 expression in DC after application of anti-TNF therapy to the bioptic specimens [52]. Silva and al. compared corticosteroid effects with those of other drugs. They proved significant decrease in CD83^+^ DC in bioptic specimens of subjects on corticosteroid therapy, while this change was not found in the subjects’ group on azathioprin [53].

Finally, this clinical study primarily served to begin with a realistic look at the CD83^+^ DC in human tissue samples and position it in a real possible clinical application. But like any clinical study, it is limited by the numerous genetic and phenotypic factors of an individual’s immune system and illness. A possible limitation of this study is the variability of the duration of the disease at the time of taking biopsy samples, given that we know that the pathophysiological mechanism of inflammation changes over time. But at the same time, this limitation can be a reason to conduct larger multi-centred studies in the future.

## 5. Conclusions

At present, the widely accepted pathophysiologic model of DC’s role in UC and CD, as two principal phenotypes of IBD, proposes that DC, which is an antigen-presenting cell, balances the response to the luminal antigen. Disturbing the balance between the tolerability and the active immunologic response is the crucial step in IBD pathogenesis. This study shows the diversity of the presence of intestinal mature CD83^+^ DC in human colon specimens according to different types of IBD, inflammation levels and therapeutic procedures. This different presence of CD83^+^ DC can be a small but possibly valuable diagnostic parameter in distinction between UC and CD, as well as a good predictor of inflammation and treatment outcome in these diseases.

## Figures and Tables

**Table 1:** Correlation between CD83^+^ DC presence (0; ≥1) and age, sex, type of disease, histopathology inflammation pattern and therapy; univariate logistic regression.

	CD83^+^ DC Presence				
	0	≥1	*p*	OR (95% CI)	*p* ^c^
Age (years)	39.5 (30–59; 15–79)	40 (31–54; 18–80)	0.889 ^a^		

Sex					
Male	32 (57)	81 (50)	0.419 ^b^		
Female	24 (43)	82 (50)			
Group of disease			0.001 ^b^		
Control †	17 (30.4)	58 (35.6)			0.002
UC	36 (64.3)	64 (39.3)		0.513 (0.26–1.0)	0.059
CD	3 (5.4)	41 (25.3)		4 (1.1–14.6)	0.035
			<0.001 ^b^		
UC †	36 (92)	64 (61)		7.8 (2.2–27)	0.001
CD	3 (8)	41 (39)			
			0.003 ^b^		
Histopathology inflammation pattern			0.049 ^b^		
Acute †	25 (44.6)	44 (27)			0.052
No infl.	21 (37.5)	79 (48.5)		2.1 (1.1–4.3)	0.030
Chronic	10 (17.9)	40 (24.5)		2.3 (0.97–5.3)	0.058
			0.086 ^b^		
Acute †	25 (71)	44 (52)			
Chronic	10 (29)	40 (48)			
			0.022 ^b^		
No infl. Chronic	31 (55)	119 (73)		2.2 (1.2–4.1)	0.015
Acute †	25 (45)	44 (27)			
Therapy			0.001 ^b^		
No †	51 (91)	111 (68)		4.8 (1.8–12.7)	0.002
Yes	5 (9)	52 (32)			

Continuous data are presented as the median (interquartile range, min–max), and categorical data are presented as the number (percentage). ^a^ Mann–Whitney U test, ^b^ χ^2^ test, ^c^ logistic regression, † reference level.

**Table 2:** Multivariate logistic regression for DC presence.

Independent Variables		OR (95% CI)	*p* ^a^
Subjects’ groups	Control and UC †	5.2 (1.4–18.6)	0.011
	CD		
Histopathology	No inflammation and chronic	2.6 (1.3–5.2)	0.005
	Acute †		
Therapy	No †	3.7 (1.3–10.2)	0.012
	Yes		

^a^ logistic regression, † reference level.

**Table 3:** Number of CD83^+^ DC according to the type of disease.

	UC (n = 100)	CD (n = 44)	*p*
CD83^+^ DS > 0.5	64 (64)	41 (93)	0.001 ^a^
CD83^+^ DS ≤ 0.5	36 (36)	3 (7)	

Categorical data are presented as the number (percentage). ^a^ χ^2^ test.

**Table 4:** Correlation between CD83^+^ DC presence (0; ≥1) and age, sex, MES, histopathology, calprotectin and therapy in UC subjects; univariate logistic regression.

CD83^+^ DC Presence					
	0	≥1	*p*	OR (95% CI)	*p* ^c^
Age (years)	44 (31–61.5; 15–79)	40 (32–55; 19–75)	0.530 ^a^		

Sex					
Male	20 (55)	30 (47)	0.532 ^b^		
Female	16 (44)	34 (54)			
MES					
Inactive disease and mild activity MES 0, 1	7 (19.4)	34 (53)	0.002 ^b^	4.7 (1.8–12.2)	0.002
Moderate activity and severe activity MES 2, 3 †	29 (81)	30 (47)			
Histopathology					
No inflammation †	3 (8.3)	12 (18.8)	0.079 ^b^		
Chronic	9 (25)	24 (37.5)			
Acute	24 (66.7)	28 (43.8)			
					
No inflammation/chronic inflammation	12 (33)	28 (44)	0.046 ^b^	2.6 (1.1–6)	0.030
Acute †	24 (67)	36 (50)			
Calprotectin					
12–146	6 (16.7)	19 (29.7)	0.555 ^b^		
147–550	10 (27.8)	15 (23.4)			
551–1799	6 (16.7)	9 (14.1)			
1800	14 (38.9)	21 (32.8)			
Therapy					
No †	31 (86)	37 (58)	0.007 ^b^	4.5 (1.6–13)	0.006
Yes	5 (14)	27 (42)			

Continuous data are presented as the median (interquartile range, min–max), and categorical data are presented as the number (percentage). ^a^ Mann–Whitney U test, ^b^ χ^2^ test, ^c^ logistic regression, † reference level.

**Table 5:** CD83^+^ DC presence in UC subjects; multivariate logistic regression.

Independent Variables		OR (95% CI)	*p* ^a^
MES-PHD	0 †	5 (1.4–17.5)	0.012
	1		
			
Therapy	No †	2.2 (0.61–8.5)	0.229
	Yes		

^a^ logistic regression, † reference level.

**Table 6:** Analysis of UC subjects with different therapy.

	Healthy Subjects	Therapy			
		No Therapy	Azathioprine	Anti TNF	
CD83^+^ DC					0.004 ^a^
0	17 (23)	31 (46)	2 (13)	3 (18)	
≥1	58 (77)	36 (54)	13 (87)	14 (82)	

Categorical data are presented as the number (percentage). ^a^ χ^2^ test.

**Table 7:** Correlation between CD83^+^ DC presence (≤1, >1) and age, sex, SES-CD, histopathology, calprotectin and therapy in CD subjects; univariate logistic regression.

CD83^+^ DC Presence					
	≤1	>1	*p*	OR (95% CI)	*p* ^c^
Age (years)	36.5 (29–42; 15–79)	38.6 (27–52; 18–64)	0.879 ^a^		

Sex					
Male	7 (39)	13 (50)	0.176 ^b^		
Female	11 (61)	13 (50)			
SES-CD					
≤5.9	4 (22)	18 (69)	0.006 ^b^	7.9 (2–31)	0.004
>5.9 †	14 (78)	8 (31)			
Histopathology					
No inflammation †	1 (5.6)	9 (34.6)	0.004 ^b^		
Chronic	5 (27.8)	12 (46.2)			
Acute	12 (66.7)	5 (19.2)			
					
No inflammation/chronic inflammation	6 (33)	21 (81)	0.004 ^b^	8.4 (2.1–33)	0.003
Acute †	12 (67)	5 (9)			
Calprotectin					
≤449	4 (22)	18 (69)	0.006 ^b^	7.9 (2–31)	0.004
>449 †	14 (78)	8 (31)			
Therapy					
No	15 (83)	4 (15)			
Yes	3 (17)	22 (85)			

Continuous data are presented as the median (interquartile range, min–max), and categorical data are presented as the number (percentage). ^a^ Mann–Whitney U test, ^b^ χ^2^ test, ^c^ logistic regression, † reference level.

**Table 8:** Analysis of CD subjects with different therapy.

	Healthy Subjects	Therapy			
		No Therapy	Azathioprine	Anti TNF	
CD83^+^ DC					<0.001 ^a^
≤1	40 (53)	15 (79)	3 (23)	0 (0)	
>1	35 (47)	4 (21)	10 (77)	12 (100)	

Categorical data are presented as the number (percentage). ^a^ χ^2^ test.

## Data Availability

We disclose no restrictions on the availability of data, materials and associated protocols.

## References

[1] De Preter V. (2015). Metabolomics in the Clinical Diagnosis of Inflammatory Bowel Disease. Dig. Dis..

[2] Dupaul-Chicoine J., Dagenais M., Saleh M. (2013). Crosstalk between the intestinal microbiota and the innate immune system in intestinal homeostasis and inflammatory bowel disease. Inflamm. Bowel Dis..

[3] Strober W., Fuss I., Mannon P. (2007). The fundamental basis of inflammatory bowel disease. J. Clin. Investig..

[4] Niess J.H. (2008). Role of mucosal dendritic cells in inflammatory bowel disease. World J. Gastroenterol..

[5] Levine A., Griffiths A., Markowitz J., Wilson D.C., Turner D., Russell R.K., Fell J., Ruemmele F.M., Walters T., Sherlock M. (2011). Pediatric modification of the Montreal classification for inflammatory bowel disease: The Paris classification. Inflamm. Bowel Dis..

[6] Kaser A., Zeissig S., Blumberg R.S. (2010). Inflammatory bowel disease. Annu. Rev. Immunol..

[7] Pavli P., Woodhams C.E., Doe W.F., Hume D.A. (1990). Isolation and characterization of antigen-presenting dendritic cells from the mouse intestinal lamina propria. Immunology.

[8] Liu L.M., MacPherson G.G. (1995). Rat intestinal dendritic cells: Immunostimulatory potency and phenotypic characterization. Immunology.

[9] Pavli P., Hume D.A., Van De Pol E., Doe W.F. (1993). Dendritic cells, the major antigen-presenting cells of the human colonic lamina propria. Immunology.

[10] Moghaddami M., Cummins A., Mayrhofer G. (1998). Lymphocyte-filled villi: Comparison with other lymphoid aggregations in the mucosa of the human small intestine. Gastroenterology.

[11] Bell S.J., Rigby R., English N., Mann S.D., Knight S.C., Kamm M.A., Stagg A.J. (2001). Migration and maturation of human colonic dendritic cells. J. Immunol..

[12] Velde A.A.T., van Kooyk Y., Braat H., Hommes D.W., Dellemijn T.A., Slors J.F.M., van Deventer S.J.H., Vyth-Dreese F.A. (2003). Increased expression of DC-SIGN+IL-12+IL-18+ and CD83+IL-12-IL-18- dendritic cell populations in the colonic mucosa of patients with Crohn’s disease. Eur. J. Immunol..

[13] Dieu M.-C., Vanbervliet B., Vicari A., Bridon J.-M., Oldham E., Aït-Yahia S., Brière F., Zlotnik A., Lebecque S., Caux C. (1998). Selective recruitment of immature and mature dendritic cells by distinct chemokines expressed in different anatomic sites. J. Exp. Med..

[14] Zhao X., Sato A., Cruz C.S.D., Linehan M., Luegering A., Kucharzik T., Shirakawa A.-K., Marquez G., Farber J.M., Williams I. (2003). CCL9 is secreted by the follicle-associated epithelium and recruits dome region Peyer’s patch CD11b+ dendritic cells. J. Immunol..

[15] Kaisho T., Akira S. (2003). Regulation of dendritic cell function through toll-like receptors. Curr. Mol. Med..

[16] Didierlaurent A., Sirard J.-C., Kraehenbuhl J.-P., Neutra M.R. (2002). How the gut senses its content. Cell Microbiol..

[17] Barton G.M., Medzhitov R. (2002). Toll-like receptors and their ligands. Curr. Top. Microbiol. Immunol..

[18] Gewirtz A.T. (2003). Intestinal epithelial toll-like receptors: To protect. And serve?. Curr. Pharm. Des..

[19] Kagnoff M.F., Eckmann L. (1997). Epithelial cells as sensors for microbial infection. J. Clin. Investig..

[20] Kapsenberg M.L. (2003). Dendritic-cell control of pathogen-driven T-cell polarization. Nat. Rev. Immunol..

[21] Sousa C.R.E., Diebold S., Edwards A., Rogers N., Schulz O., Spörri R. (2003). Regulation of dendritic cell function by microbial stimuli. Pathol. Biol..

[22] Shortman K., Liu Y.J. (2002). Mouse and human dendritic cell subtypes. Nat. Rev. Immunol..

[23] Mazzoni A., Segal D.M. (2004). Controlling the Toll road to dendritic cell polarization. J. Leukoc. Biol..

[24] Rescigno M., Di Sabatino A. (2009). Dendritic cells in intestinal homeostasis and disease. J. Clin. Investig..

[25] Stange E., Travis S., Vermeire S., Reinisch W., Geboes K., Barakauskiene A., Feakins R., Fléjou J., Herfarth H., Hommes D. (2008). European evidence-based Consensus on the diagnosis and management of ulcerative colitis: Definitions and diagnosis. J. Crohns Colitis.

[26] Dignass A., Eliakim R., Magro F., Maaser C., Chowers Y., Geboes K., Mantzaris G., Reinisch W., Colombel J.F., Vermeire S. (2012). Second European evidence-based consensus on the diagnosis and management of ulcerative colitis part 1, definitions and diagnosis. J. Crohns Colitis.

[27] Maaser C., Sturm A., Vavricka S.R., Kucharzik T., Fiorino G., Annese V., Calabrese E., Baumgart D.C., Bettenworth D., Borralho Nunes P. (2018). ECCO-ESGAR Guideline for Diagnostic Assessment in IBD Part 1, Initial diagnosis, monitoring of known IBD, detections of complications. J. Crohns Colitis.

[28] Van Assche G., Dignass A., Panes J., Beaugerie L., Karagiannis J., Allez M., Rogler G., Louis E., Kupcinskas L., Mantzaris G. (2010). European evidence-based consensus on the diagnosis and management of Crohn’s disease: Definitions and diagnosis. J. Crohns Colitis.

[29] Gomollón F., Dignass A., Annese V., Tilg H., Van Assche G., Lindsay J.O., Peyrin-Biroulet L., Cullen G.J., Daperno M., Kucharzik T. (2017). 3rd European Evidence-based Consensus on the Diagnosis and Management of Crohn’s Disease 2016: Part 1, Diagnosis and Medical Management. J. Crohns Colitis.

[30] Schroeder K.W., Tremaine W.J., Ilstrup D.M. (1987). Coated oral 5-aminosalicylic acid therapy for mildly to moderately active ulcerative colitis. A randomized study. N. Engl. J. Med..

[31] Annese V., Daperno M., Rutter M.D., Amiot A., Bossuyt P., East J., Ferrante M., Götz M., Katsanos K.H., Kießlich R. (2013). European evidence based consensus for endoscopy in inflammatory bowel disease. J. Crohns Colitis.

[32] Daperno M., D’Haens G., Van Assche G., Baert F., Bulois P., Maunoury V., Sostegni R., Rocca R., Pera A., Gevers A. (2004). Development and validation of a new, simplified endoscopic activity score for Crohn’s disease: The SES-CD. Gastrointest Endosc..

[33] Sipponen T., Nuutinen H., Turunen U., Färkkilä M. (2010). Endoscopic evaluation of Crohn’s disease activity: Comparison of the CDEIS and the SES-CD. Inflamm. Bowel Dis..

[34] Middel P., Raddatz D., Gunawan B., Haller F., Radzun H.J. (2006). Increased number of mature dendritic cells in Crohn’s disease: Evidence for a chemokine mediated retention mechanism. Gut.

[35] Baumgart D.C., Sandborn W.J. (2007). Inflammatory bowel disease: Clinical aspects and established and evolving therapies. Lancet.

[36] Baumgart D.C., Metzke D., Schmitz J., Scheffold A., Sturm A., Wiedenmann B., Dignass A.U. (2005). Patients with active inflammatory bowel disease lack immature peripheral blood plasmacytoid and myeloid dendritic cells. Gut.

[37] Radwan-Kwiatek K., Tabarkiewicz J., Radwan P., Rolinski J. (2007). CD1c+/CD19- and CD303+/CD123+ dendritic cells in the peripheral blood in patients with ulcerative colitis and Crohn’s disease. Pol. J. Environ. Stud..

[38] Radwan-Kwiatek K., Radwan P., Tatarkiewicz J., Rolinski J. (2009). Circulating dendritic cells as a novel disease activity marker in inflammatory bowel disease?. Gut.

[39] Bates J., Flanagan K., Mo L., Ota N., Ding J., Ho S., Liu S., Roose-Girma M., Warming S., Diehl L. (2015). Dendritic cell CD83 homotypic interactions regulate inflammation and promote mucosal homeostasis. Mucosal Immunol..

[40] Silva M.A., López C.B., Riverin F., Oligny L., Menezes J., Seidman E.G. (2004). Characterization and distribution of colonic dendritic cells in Crohn’s disease. Inflamm. Bowel Dis..

[41] Kawashima D., Oshitani N., Jinno Y., Watanabe K., Nakamura S., Higuchi K., Arakawa T. (2005). Augmented expression of secondary lymphoid tissue chemokine and EBI1 ligand chemokine in Crohn’s disease. J. Clin. Pathol..

[42] Baumgart D.C., Thomas S., Przesdzing I., Metzke D., Bielecki C., Lehmann S.M., Lehnardt S., Dörffel Y., Sturm A., Scheffold A. (2009). Exaggerated inflammatory response of primary human myeloid dendritic cells to lipopolysaccharide in patients with inflammatory bowel disease. Clin. Exp. Immunol..

[43] Røseth A.G., Aadland E., Grzyb K. (2004). Normalization of faecal calprotectin: A predictor of mucosal healing in patients with inflammatory bowel disease. Scand. J. Gastroenterol..

[44] Vieira A., Fang C.B., Rolim E.G., Klug W.A., Steinwurz F., Rossini L.G.B., Candelária P.A. (2009). Inflammatory bowel disease activity assessed by fecal calprotectin and lactoferrin: Correlation with laboratory parameters, clinical, endoscopic and histological indexes. BMC Res. Notes.

[45] D’incà R., Pont E.D., Di Leo V., Ferronato A., Fries W., Vettorato M.G., Martines D., Sturniolo G.C. (2007). Calprotectin and lactoferrin in the assessment of intestinal inflammation and organic disease. Int. J. Colorectal. Dis..

[46] Kaiser T., Langhorst J., Wittkowski H., Becker K., Friedrich A.W., Rueffer A., Dobos G.J., Roth J., Foell D. (2007). Faecal S100A12 as a non invasive marker distinguishing inflammatory bowel disease from irritable bowel syndrome. Gut.

[47] Bodelier A.G.L., Jonkers D., Heuvel T.v.D., de Boer E., Hameeteman W., Masclee A.A.M., Pierik M.J. (2017). High percentage of IBD patients with indefinite fecal calprotectin levels: Additional value of a combination score. Dig. Dis. Sci..

[48] D’haens G., Ferrante M., Vermeire S., Baert F., Noman M., Moortgat L., Geens P., Iwens D., Aerden I., Van Assche G. (2012). Fecal calprotectin is a surrogate marker for endoscopic lesions in inflammatory bowel disease. Inflamm. Bowel Dis..

[49] Mosli M.H., Zou G., Garg S.K., Feagan S.G., MacDonald J.K., Chande N., Sandborn W.J., Feagan B.G. (2015). C-reactive protein, fecal calprotectin, and stool lactoferrin for detection of endoscopic activity in symptomatic inflammatory bowel disease patients: A systematic review and meta-analysis. Am. J. Gastroenterol..

[50] Duchmann R., Kaiser I., Hermann E., Mayet W., Ewe K., Büschenfelde K.H.M.Z. (1995). Tolerance exists towards resident intestinal flora but is broken in active inflammatory bowel disease (IBD). Clin. Exp. Immunol..

[51] Bhandaru M., Pasham V., Yang W., Bobbala D., Rotte A., Lang F. (2012). Effect of azathioprine on Na^+^/H^+^ exchanger activity in dendritic cells. Cell Physiol. Biochem..

[52] Hart A.L., Al-Hassi H.O., Rigby R.J., Bell S.J., Emmanuel A.V., Knight S.C., Kamm M.A., Stagg A.J. (2005). Characteristics of intestinal dendritic cells in inflammatory bowel diseases. Gastroenterology.

[53] Silva M.A., Quera R., Valenzuela J., Salim S.A.Y., Söderholm J.D., Perdue M.H. (2008). Dendritic cells and toll-like receptors 2 and 4 in the ileum of Crohn’s disease patients. Dig. Dis. Sci..

